# Mechanical Response of MEMS Inductor with Auxiliary Pillar under High-g Shock

**DOI:** 10.3390/mi9040176

**Published:** 2018-04-11

**Authors:** Lixin Xu, Yiyuan Li, Jianhua Li, Chongying Lu

**Affiliations:** 1 School of Mechatronical Engineering, Beijing Institute of Technology, Beijing 100081, China; lxxu@bit.edu.cn (L.X.); lyy510@bit.edu.cn (Y.L.); 2 TaiYuan Satellite Launch Center, Xinzhou 30027, China; luchongying@163.com

**Keywords:** MEMS suspended inductor, high mechanical shock, MEMS reliability, response analysis

## Abstract

Micro-electromechanical system (MEMS) suspended inductors have excellent radio-frequency (RF) performance, but poor mechanical properties. To improve their reliability, auxiliary pillars have been used. However, few studies have been carried out on the response of a suspended inductor with auxiliary pillars under high mechanical shock. In this paper, a theoretical method is proposed that combines a single-degree-of-freedom (SDOF) model and a method for solving statically indeterminate structures. The calculated results obtained by this proposed method were verified by finite-element analysis (ANSYS). The calculated results obtained by the proposed method were found to agree well with the results of ANSYS simulation. Finally, this method was extended to a suspended inductor with double auxiliary pillars. The method proposed in this paper provides a theoretical reference for mechanical performance evaluation and reliability optimization design for MEMS suspended inductors with auxiliary pillars.

## 1. Introduction

Micro-electromechanical system (MEMS) suspended inductors show excellent radio-frequency (RF) performance because they lift the inductor coil several micrometers above the substrate [1,2,3]. However, MEMS suspended inductors have poor mechanical properties. They tend to fail under mechanical shock during fabrication, shipping, storage, and operation. In particular, in military applications, the suspended inductor must withstand high mechanical shocks of amplitudes in the order of 10^4^–10^5^ g [4]. Such a high mechanical shock will cause deformation or even failure of the MEMS suspended inductor. To improve the reliability of MEMS suspended inductors, several studies have been carried out. Hsieh et al. [5] used a Si_3_N_4_/SiO_2_ X-beam to increase the mechanical strength of the suspended inductor coil. Lin et al. [6] and Ribas et al. [7] designed two kinds of suspended inductors with a sandwich dielectric membrane support to suspend the inductor coil. Wang et al. [8] designed a suspended inductor with auxiliary pillars. Compared with an inductor with dielectric membrane, the inductor with auxiliary pillars can achieve a higher suspension height to reduce substrate loss and achieve a higher quality factor.

Many theoretical studies have been carried out on the dynamic response of MEMS devices under mechanical shock. However, most of these studies were focused on devices with simple structures, such as micro-beams, MEMS switches, etc. [9,10,11,12]. As for MEMS with complex structures such as suspended inductors and accelerometers, it is difficult to model them and to solve for their dynamic responses under mechanical shock. Srikar et al. [13] analyzed the mechanical responses of a large class of shock-loaded MEMS and modeled their microstructures using an undamped single-degree-of-freedom (SDOF) model attached to an accelerating base. Li et al. [14] studied the motion of MEMS accelerometers during drop tests. They used an SDOF model and a continuous beam model to take the flexibility of the microstructures into account and to calculate their maximum deflection. Sundaram et al. [15] developed a combined experimental–analytical approach to predict the failure of MEMS devices. They used an SDOF model to solve for the displacement of the moving structures under mechanical shock and then obtained the critical acceleration for failure. However, few investigations have been carried out on the response of MEMS suspended inductors with auxiliary pillars under mechanical shock.

This paper presents a method that can be used to model the response of MEMS suspended inductor with auxiliary pillar under high-g shock. The deformation and stress of the inductor can be calculated using the parameters of the inductor geometry and the shock pulse. First, the mechanical response of the suspended inductor with auxiliary pillar under mechanical shock is analyzed, and the deformations and equivalent stresses are formulated. Then the ANSYS finite-element (FE) software is used to verify the results calculated by the method. Finally, the method is extended to the suspended inductor with double auxiliary pillars.

## 2. Mechanical Response of a Suspended Inductor with Auxiliary Pillar under Shock

### 2.1. Model of the MEMS Suspended Inductor with Auxiliary Pillar and Shock Loads

**Figure 1:**
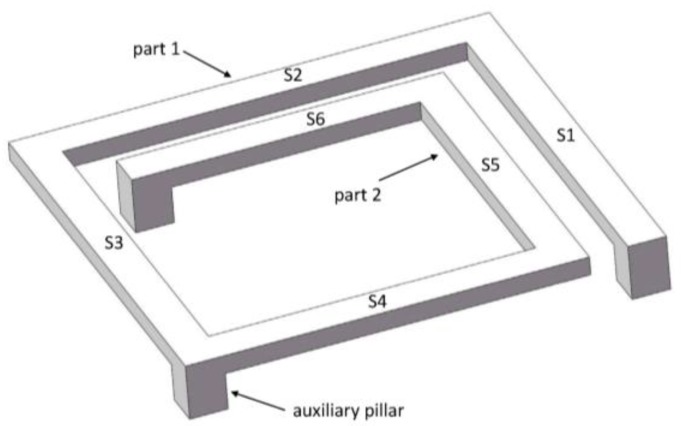
Schematic of the MEMS suspended inductor with auxiliary pillar.

**Figure 2:**
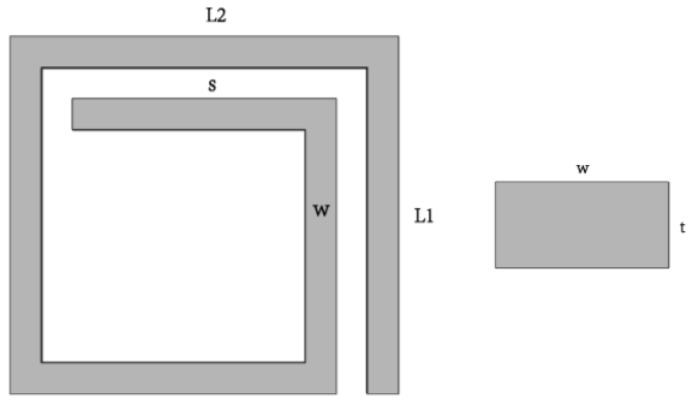
Parameters of the suspended inductor.

Figure 1 shows the model of a MEMS suspended inductor with auxiliary pillar used in this study. The inductor coil is composed of six segments, and the wire segments are denoted by S1–S6. We refer to the inductor in the previous study [16] to decide the parameters of the inductor we use in this study. The parameters of the inductor have been analyzed and optimized with the electromagnetic finite element analysis software HFSS in [16]. Figure 2 gives the parameters of the inductor coil: the wire width (w), spacing (s), and thickness (t) are 20 μm, 20 μm, and 10 μm, respectively. The lengths of L1 and L2 are 210 μm and 230 μm, respectively. The suspended height is 20 μm.

**Figure 3:**
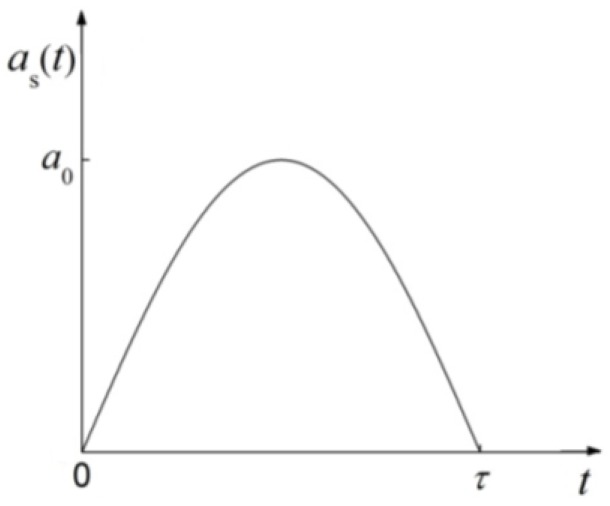
Half-sine acceleration pulse.

The half-sine waveform is a typical shock pulse that is widely used in theoretical analysis. It provides a good description of the acceleration observed in several tests, such as the air cannon and the Hopkinson bar [13,17,18]. The half-sine waveform can be expressed as:

(1) $$a_{s}\left(t\right)=\{\begin{array}{ll} a_{0}\sin\left(\frac{\pi t}{\tau }\right) & 0\leq t\leq \tau \\ 0 & \tau <t \end{array},$$

where $a_{0}$ is the amplitude of the shock and τ is the duration of the shock load. As shown in Figure 3, half-sine shock loads can be characterized by specifying the amplitude and the duration.

### 2.2. Acceleration Response of the Suspended Inductor with Auxiliary Pillar

**Figure 4:**
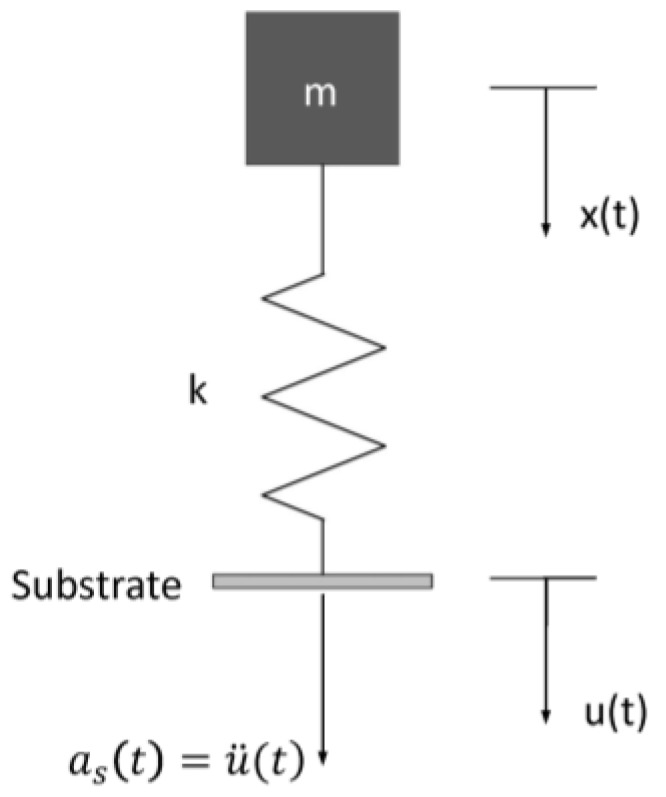
SDOF model of the MEMS suspended inductor.

Generally, the silicon substrate is considered as a rigid body under mechanical shock, and the inductor coil can be modeled as a resonator attached to an accelerating support [13]. The resonator is considered as a linear and undamped spring-mass system with a single degree of freedom. As for the suspended inductor with auxiliary pillar in Figure 1, the inductor coil is divided into two parts by the pillar. Neglecting the deformation of the pillar, each part of the coil can be modeled as an undamped SDOF system, as shown in Figure 4.

The mass and the spring constant of each part of the coil are denoted by *m* and *k*, respectively. *x*(*t*) and *u*(*t*) are the absolute displacement of the mass and the substrate, respectively.

The equation of mass motion can be expressed as:

(2) $$\frac{m\ddot{x}}{k}+x\left(t\right)=u\left(t\right)$$

Equation (2) can also be expressed as:

(3) $$\frac{m}{k}\cdot \frac{d^{2}\ddot{x}}{dt^{2}}+\ddot{x}=\ddot{u}\left(t\right)$$

and

(4) $$\frac{1}{\omega_{n}{}^{2}}\cdot \ddot{a}+a=a_{s}\left(t\right)$$

In Equation (4), *a* is the absolute acceleration response of the mass, $a_{s}\left(t\right)$ is the acceleration load applied on the substrate, and $\omega_{n}$ is the natural angular frequency.

Equation (4) can be solved using the Laplace transform [19]. The absolute acceleration response of the mass is:

(5) $$a\left(t\right)=\{\begin{array}{cc} a_{0}\cdot \frac{\frac{T}{2\tau }\sin\omega_{n}t-\sin\frac{\pi }{\tau }t}{\frac{T^{2}}{4\tau^{2}}-1} & 0\leq t\leq \tau \\ \;a_{0}\cdot \frac{\frac{T}{\tau }\cos\left(\frac{\pi \tau }{T}\right)}{\frac{T^{2}}{4\tau^{2}}-1}\;\sin\omega_{n}\left(t-\frac{\tau }{2}\right) & \tau <t \end{array}$$

where *T* is the time period of vibration and is given by $T=2\pi /\omega_{n}$.

As shown in Equation (5), the absolute acceleration response of the mass is determined by the time period of vibration *T*, the natural angular frequency $\omega_{n}$, the amplitude of the shock $a_{0}$, and the duration of the load τ. T and $\omega_{n}$ of the structure can be obtained by modal analysis.

### 2.3. Mechanical Response of the Suspended Inductor with Auxiliary Pillar under Mechanical Shock

**Figure 5:**
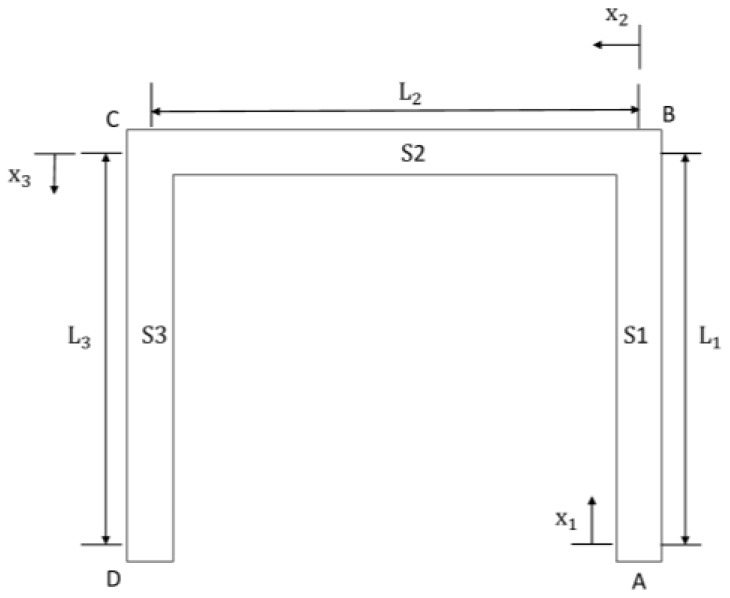
Top view of Part 1 of the coil.

For the suspended inductor with auxiliary pillar in Figure 1, the inductor coil is divided into two bending structures by the auxiliary pillar. Each part is a statically indeterminate structure because the deformation of the pillar can be ignored. Figure 5 shows a top view of Part 1.

The lengths of the three wire segments are $L_{1}$, $L_{2}$, and $L_{3}$, respectively. Only inertial forces caused by shock load are considered here because the influence of substrate deformation and traveling elastic waves can be ignored [13]. The inertial distribution load of the *i*-th wire segment *S_i_* under shock can be expressed by:

(6) $$q_{i}=w_{i}t_{i}\rho a$$

where ρ is the density of copper, *a* is the absolute acceleration response, and $w_{i}$ and $t_{i}$ are the width and thickness, respectively, of the *i*-th wire segment.

**Figure 6:**
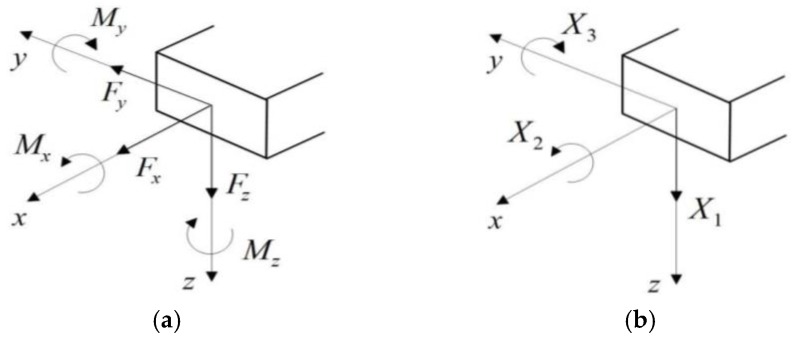
Generalized forces added on the released end.

By releasing the constraint of the clamped end A (see Figure 5) and adding six generalized forces (see Figure 6a), the equivalent system of the statically indeterminate structure can be determined. Because the shock-sensitive direction of the inductor is perpendicular to its coil plane, only shock along the *z*-direction is considered in this paper. By neglecting structural motion in the *x-y* plane, the six generalized forces can be simplified to three, as shown in Figure 6b: a force $F_{z}$ ($X_{1}$ in Figure 6b), a bending moment $M_{y}$ ($X_{3}$ in Figure 6b), and a torque $M_{x}$ ($X_{2}$ in Figure 6b).

Then the canonical equation of force method can be established:

(7) $$\left[\begin{array}{lll} \delta_{11} & \delta_{12} & \delta_{13}\\ \delta_{21} & \delta_{22} & \delta_{23}\\ \delta_{31} & \delta_{32} & \delta_{33} \end{array}\right]\cdot \left[\begin{array}{l} X_{1}\\ X_{2}\\ X_{3} \end{array}\right]+\left[\begin{array}{l} \Delta_{1F}\\ \Delta_{2F}\\ \Delta_{3F} \end{array}\right]=\left[\begin{array}{l} \Delta_{1}\\ \Delta_{2}\\ \Delta_{3} \end{array}\right]$$

where $\delta_{ij}$ is the generalized displacement of the released end along the $X_{i}$ direction, which is caused only by unit force $X_{j}$. $\Delta_{iF}$ is the generalized displacement of the released end along the $X_{i}$ direction, which is caused only by the shock load. $\Delta_{i}$ is the generalized displacement of the statically indeterminate structure along the $X_{i}$ direction. $\delta_{ij}$ and $\Delta_{iF}$ can be calculated by Mohr’s integration. $\Delta_{1}=\Delta_{2}=\Delta_{3}=0$ because the released end is a clamped end.

Equation (7) can also be expressed as:

(8) $$\left[\begin{array}{l} X_{1}\\ X_{2}\\ X_{3} \end{array}\right]=-{\left[\begin{array}{lll} \delta_{11} & \delta_{12} & \delta_{13}\\ \delta_{21} & \delta_{22} & \delta_{23}\\ \delta_{31} & \delta_{32} & \delta_{33} \end{array}\right]}^{-1}\cdot \left[\begin{array}{l} \Delta_{1F}\\ \Delta_{2F}\\ \Delta_{3F} \end{array}\right]$$

where $\Delta_{1F}$ is the generalized displacement of the released end along the $X_{1}$ direction, which is caused only by the shock load. According to Mohr’s integration, $\Delta_{1F}$ can be expressed as:

(9) $$\Delta_{1F}=\sum \int_{L_{i}}\frac{M_{qi}(x_{i})\cdot {\bar{M}}_{Fi}(x_{i})dx_{i}}{EI_{i}}+\sum \int_{L_{i}}\frac{T_{qi}(x_{i})\cdot {\bar{T}}_{Fi}(x_{i})dx_{i}}{GI_{ti}},$$

where *E* and *G* are the Young’s modulus and the shear modulus of copper, respectively. $x_{i}$ is a location indicator variable ($0<x_{i}<L_{i}$), $M_{qi}\left(x_{i}\right)$ and $T_{qi}\left(x_{i}\right)$ are the bending moment and torque of the *i*-th wire segment caused by shock load, respectively, and ${\bar{M}}_{Fi}\left(x_{i}\right)$ and ${\bar{T}}_{Fi}\left(x_{i}\right)$ are the bending moment and torque of the *i*-th wire segment caused by unit force along the $X_{1}$ direction, respectively. $I_{i}$ and $I_{ti}$ are the moment of inertia and the polar moment of inertia of the *i*-th wire segment’s cross section, respectively.

For wire segment S1:

(10) $$M_{q1}\left(x_{1}\right)=-\frac{q_{1}x_{1}{}^{2}}{2}$$

(11) $$T_{q1}\left(x_{1}\right)=0$$

(12) $${\bar{M}}_{F1}\left(x_{1}\right)=-x_{1}$$

(13) $${\bar{T}}_{F1}\left(x_{1}\right)=0$$

For wire segment S2:

(14) $$M_{q2}\left(x_{2}\right)=-\frac{q_{2}x_{2}{}^{2}}{2}-q_{1}L_{1}x_{2}$$

(15) $$T_{q2}\left(x_{2}\right)=\frac{q_{1}L_{1}{}^{2}}{2}$$

(16) $${\bar{M}}_{F2}\left(x_{2}\right)=-x_{2}$$

(17) $${\bar{T}}_{F2}\left(x_{2}\right)=L_{1}$$

For wire segment S3:

(18) $$M_{q3}\left(x_{3}\right)=-\frac{q_{3}x_{3}{}^{2}}{2}-q_{2}L_{2}x_{3}+q_{1}L_{1}\left(\frac{L_{1}}{2}-x_{3}\right)$$

(19) $$T_{q3}\left(x_{3}\right)=\frac{q_{2}L_{2}{}^{2}}{2}+q_{1}L_{1}L_{2}$$

(20) $${\bar{M}}_{F3}\left(x_{3}\right)=L_{3}-x_{3}$$

(21) $${\bar{T}}_{F3}\left(x_{3}\right)=L_{2}$$

By substituting Equations (10)–(21) into (9), $\Delta_{1F}$ can be expressed as:

(22) $$\Delta_{1F}=\frac{q_{1}L_{1}{}^{4}}{8EI_{1}}+\frac{q_{2}L_{2}{}^{4}}{8EI_{2}}+\frac{q_{1}L_{1}L_{2}{}^{3}}{3EI_{2}}-\frac{q_{3}L_{3}{}^{4}}{24EI_{3}}-\frac{q_{2}L_{2}L_{3}{}^{3}}{6EI_{3}}+\frac{q_{1}L_{1}{}^{2}L_{3}{}^{2}}{4EI_{3}}-\frac{q_{1}L_{1}L_{3}{}^{3}}{6EI_{3}}+\frac{q_{1}L_{1}{}^{3}L_{2}}{2GI_{t2}}+\frac{q_{2}L_{2}{}^{3}L_{3}}{2GI_{t3}}+\frac{q_{1}L_{1}L_{2}{}^{2}L_{3}}{GI_{t3}}$$

$\Delta_{2F}$ is the generalized displacement of the released end along the $X_{2}$ direction, which is caused only by the shock load. $\Delta_{2F}$ can be expressed as:

(23) $$\Delta_{2F}=\sum \int_{L_{i}}\frac{M_{qi}(x_{i})\cdot {\bar{M}}_{Ti}(x_{i})dx_{i}}{EI_{i}}+\sum \int_{L_{i}}\frac{T_{qi}(x_{i})\cdot {\bar{T}}_{Ti}(x_{i})dx_{i}}{GI_{ti}}$$

where ${\bar{M}}_{Ti}\left(x_{i}\right)$ and ${\bar{T}}_{Ti}\left(x_{i}\right)$ are the bending moment and torque, respectively, of the *i*-th wire segment caused by a unit generalized force along the $X_{2}$ direction.

For wire segment S1:

(24) $${\bar{M}}_{T1}\left(x_{1}\right)=0$$

(25) $${\bar{T}}_{T1}\left(x_{1}\right)=1$$

For wire segment S2:

(26) $${\bar{M}}_{T2}\left(x_{2}\right)=1$$

(27) $${\bar{T}}_{T2}\left(x_{2}\right)=0$$

For wire segment S3:

(28) $${\bar{M}}_{T3}\left(x_{3}\right)=0$$

(29) $${\bar{T}}_{T3}\left(x_{3}\right)=-1$$

By substituting Equations (10), (11), (14), (15), (18), (19), (24)–(29) into (23), $\Delta_{2F}$ can be expressed as:

(30) $$\Delta_{2F}=-\frac{q_{2}L_{2}{}^{3}}{6EI_{2}}-\frac{q_{1}L_{1}L_{2}{}^{2}}{2EI_{2}}-\frac{q_{2}L_{2}{}^{2}L_{3}}{2GI_{t3}}-\frac{q_{1}L_{1}L_{2}L_{3}}{2GI_{t3}}$$

$\Delta_{3F}$ is the generalized displacement of the released end along the $X_{3}$ direction, which is caused only by the shock load. $\Delta_{3F}$ can be expressed as:

(31) $$\Delta_{3F}=\sum \int_{L_{i}}\frac{M_{qi}(x_{i})\cdot {\bar{M}}_{Mi}(x_{i})dx_{i}}{EI_{i}}+\sum \int_{L_{i}}\frac{T_{qi}(x_{i})\cdot {\bar{T}}_{Mi}(x_{i})dx_{i}}{GI_{ti}}$$

where ${\bar{M}}_{Mi}\left(x_{i}\right)$ and ${\bar{T}}_{Mi}\left(x_{i}\right)$ are the bending moment and torque, respectively, of the *i*-th wire segment caused by a unit generalized force along the $X_{3}$ direction.

For wire segment S1:

(32) $${\bar{M}}_{M1}\left(x_{1}\right)=1$$

(33) $${\bar{T}}_{M1}\left(x_{1}\right)=0$$

For wire segment S2:

(34) $${\bar{M}}_{M2}\left(x_{2}\right)=0$$

(35) $${\bar{T}}_{M2}\left(x_{2}\right)=-1$$

For wire segment S3:

(36) $${\bar{M}}_{M3}\left(x_{3}\right)=-1$$

(37) $${\bar{T}}_{M3}\left(x_{3}\right)=0$$

By substituting Equations (10), (11), (14), (15), (18), (19), and (32)–(37) into (31), $\Delta_{3F}$ can be expressed as:

(38) $$\Delta_{3F}=-\frac{q_{1}L_{1}{}^{3}}{6EI_{1}}+\frac{q_{3}L_{3}}{6EI_{3}}+\frac{q_{2}L_{2}L_{3}{}^{3}}{2EI_{3}}-\frac{q_{1}L_{1}{}^{2}L_{3}}{2EI_{3}}+\frac{q_{1}L_{1}L_{3}{}^{2}}{2EI_{3}}-\frac{q_{1}L_{1}{}^{2}L_{2}}{2GI_{t2}}$$

$\delta_{ij}$ is the generalized displacement of the released end along the $X_{i}$ direction, which is caused only by $X_{j}$ and $X_{j}=1$. According to Mohr’s integration, $\delta_{ij}$ can be expressed as follows:

(39) $$\begin{array}{ll} \delta_{11} & =\sum \int_{L_{i}}\frac{{\bar{M}}_{Fi}(x_{i})\cdot {\bar{M}}_{Fi}(x_{i})dx_{i}}{EI_{i}}+\sum \int_{L_{i}}\frac{{\bar{T}}_{Fi}(x_{i})\cdot {\bar{T}}_{Fi}(x_{i})dx_{i}}{GI_{ti}}\\ & =\frac{L_{1}{}^{3}}{3EI_{1}}+\frac{L_{2}{}^{3}}{3EI_{2}}+\frac{L_{3}{}^{3}}{3EI_{3}}+\frac{L_{1}{}^{2}L_{2}}{GI_{t2}}+\frac{L_{2}{}^{2}L_{3}}{GI_{t3}} \end{array}$$

(40) $$\delta_{12}=\delta_{21}=\sum \int_{L_{i}}\frac{{\bar{M}}_{Ti}(x_{i})\cdot {\bar{M}}_{Fi}(x_{i})dx_{i}}{EI_{i}}+\sum \int_{L_{i}}\frac{{\bar{T}}_{Ti}(x_{i})\cdot {\bar{T}}_{Fi}(x_{i})dx_{i}}{GI_{ti}}=-\frac{L_{2}{}^{3}}{2EI_{2}}-\frac{L_{2}L_{3}}{GI_{t3}}$$

(41) $$\begin{array}{ll} \delta_{13}=\delta_{31} & =\sum \int_{L_{i}}\frac{{\bar{M}}_{Mi}(x_{i})\cdot {\bar{M}}_{Fi}(x_{i})dx_{i}}{EI_{i}}+\sum \int_{L_{i}}\frac{{\bar{T}}_{Mi}(x_{i})\cdot {\bar{T}}_{Fi}(x_{i})dx_{i}}{GI_{ti}}\\ & =-\frac{L_{1}{}^{3}}{2EI_{1}}-\frac{L_{3}{}^{3}}{2EI_{3}}-\frac{L_{1}L_{2}}{GI_{t2}} \end{array}$$

(42) $$\delta_{22}=\sum \int_{L_{i}}\frac{{\bar{M}}_{Ti}(x_{i})\cdot {\bar{M}}_{Ti}(x_{i})dx_{i}}{EI_{i}}+\sum \int_{L_{i}}\frac{{\bar{T}}_{Ti}(x_{i})\cdot {\bar{T}}_{Ti}(x_{i})dx_{i}}{GI_{ti}}=\frac{L_{2}}{EI_{2}}+\frac{L_{1}}{GI_{t1}}+\frac{L_{3}}{GI_{t3}}$$

(43) $$\delta_{23}=\delta_{32}=\sum \int_{L_{i}}\frac{{\bar{M}}_{Mi}(x_{i})\cdot {\bar{M}}_{Ti}(x_{i})dx_{i}}{EI_{i}}+\sum \int_{L_{i}}\frac{{\bar{T}}_{Mi}(x_{i})\cdot {\bar{T}}_{Ti}(x_{i})dx_{i}}{GI_{ti}}=0$$

(44) $$\delta_{33}=\sum \int_{L_{i}}\frac{{\bar{M}}_{Mi}(x_{i})\cdot {\bar{M}}_{Mi}(x_{i})dx_{i}}{EI_{i}}+\sum \int_{L_{i}}\frac{{\bar{T}}_{Mi}(x_{i})\cdot {\bar{T}}_{Mi}(x_{i})dx_{i}}{GI_{ti}}\;=\frac{L_{1}}{EI_{1}}+\frac{L_{3}}{EI_{3}}+\frac{L_{2}}{GI_{t2}}$$

By substituting Equations (22), (30), (38), and (39)–(44) into (8), one can solve for the generalized forces $X_{1}$, $X_{2}$, and $X_{3}$. Then the bending moment $M_{i}\left(x_{i}\right)$ and the torque $T_{i}\left(x_{i}\right)$ of the structure under shock can be calculated.

For wire segment S1:

(45) $$M_{1}\left(x_{1}\right)=-\frac{q_{1}x_{1}{}^{2}}{2}-X_{1}x_{1}+X_{3}$$

(46) $$T_{1}\left(x_{1}\right)=X_{2}$$

For wire segment S2:

(47) $$M_{2}\left(x_{2}\right)=-\frac{q_{2}x_{2}{}^{2}}{2}-q_{1}L_{1}x_{2}-X_{1}x_{2}+X_{2}$$

(48) $$T_{2}\left(x_{2}\right)=\frac{q_{1}L_{1}{}^{2}}{2}+X_{1}L_{1}-X_{3}$$

For wire segment S3:

(49) $$M_{3}\left(x_{3}\right)=-\frac{q_{3}x_{3}{}^{2}}{2}-q_{2}L_{2}x_{3}+\frac{q_{1}L_{1}{}^{2}}{2}-q_{1}L_{1}x_{3}+X_{1}\left(L_{3}-x_{3}\right)-X_{3}$$

(50) $$T_{3}\left(x_{3}\right)=\frac{q_{2}L_{2}{}^{2}}{2}+q_{1}L_{1}L_{2}+X_{1}L_{2}-X_{2}$$

**Figure 7:**
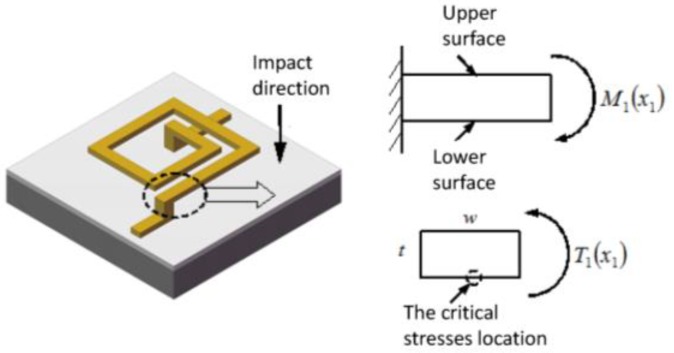
Critical stress location of the inductor coil.

When the suspended inductor coil is subjected to a mechanical shock load perpendicular to the coil plane, both the critical normal stress and the critical shear stress will appear on the midpoint of the w side of the coil wire cross section, as shown in Figure 7.

The critical normal stress of the *i*-th wire segment can be expressed as:

(51) $$\sigma_{i-max}\left(x_{i}\right)=\frac{M_{i}\left(x_{i}\right)}{I_{i}}\cdot \frac{t_{i}}{2}$$

The critical shear stress of the *i*-th wire segment can be expressed as:

(52) $$\tau_{i-max}\left(x_{i}\right)=\frac{T_{i}\left(x_{i}\right)}{\alpha_{i}w_{i}t_{i}{}^{2}}$$

The critical normal stress and the critical shear stress of the *i*-th wire segment can be calculated by substituting Equations (45)–(50) into (51) and (52).

The Von Mises equivalent stress of the *i*-th wire segment can be expressed as:

(53) $$\sigma_{i-VonMises}\left(x_{i}\right)=\sqrt{\sigma_{i-max}{\left(x_{i}\right)}^{2}+3\tau_{i-max}{\left(x_{i}\right)}^{2}}$$

The deformation at any position of wire segment S1 can be expressed as:

(54) $$d_{1}(a_{1})=\int_{a_{1}}\frac{M_{1}(x_{1})\cdot {\bar{M}}_{F1}(x_{1})dx_{1}}{EI_{1}}+\int_{a_{1}}\frac{T_{1}(x_{1})\cdot {\bar{T}}_{F1}(x_{1})dx_{1}}{GI_{t1}}$$

where

(55) $${\bar{M}}_{F1}\left(x_{1}\right)=-\left(a_{1}-x_{1}\right)$$

(56) $${\bar{T}}_{F1}\left(x_{1}\right)=0$$

where $a_{1}$ is a position indicator variable, $0\leq a_{1}\leq L_{1}$.

By substituting Equations (45), (46), (55) and (56) into (54), the deformation at any position of S1 can be expressed as:

(57) $$d_{1}\left(a_{1}\right)=\frac{1}{EI_{1}}\left(\frac{q_{1}a_{1}{}^{4}}{24}+\frac{X_{1}a_{1}{}^{3}}{6}-\frac{X_{3}a_{1}{}^{2}}{2}\right)$$

The deformation at any position of S2 can be expressed as:

(58) $$d_{2}(a_{2})=\sum \int_{L_{i}}\frac{M_{i}(x_{i})\cdot {\bar{M}}_{Fi}(x_{i})dx_{i}}{EI_{i}}+\sum \int_{L_{i}}\frac{T_{i}(x_{i})\cdot {\bar{T}}_{Fi}(x_{i})dx_{i}}{GI_{ti}}$$

where

(59) $${\bar{M}}_{F1}\left(x_{1}\right)=-\left(L_{1}-x_{1}\right)$$

(60) $${\bar{T}}_{F1}\left(x_{1}\right)=-a_{2}$$

(61) $${\bar{M}}_{F2}\left(x_{2}\right)=-\left(a_{2}-x_{2}\right)$$

(62) $${\bar{T}}_{F2}\left(x_{2}\right)=0$$

where $a_{2}$ is a position indicator variable, $0\leq a_{2}\leq L_{2}$.

By substituting Equations (45)–(48) and (59)–(62) into (58), the deformation at any position of S2 can be expressed as:

(63) $$d_{2}\left(a_{2}\right)=\frac{1}{EI_{1}}\left(\frac{q_{1}L_{1}{}^{4}}{24}+\frac{X_{1}L_{1}{}^{3}}{6}-\frac{X_{3}L_{1}{}^{2}}{2}\right)+\frac{1}{EI_{2}}\left(\frac{q_{2}a_{2}{}^{4}}{24}+\frac{q_{1}L_{1}a_{2}{}^{3}}{6}+\frac{X_{1}a_{2}{}^{3}}{6}-\frac{X_{2}a_{2}{}^{2}}{2}\right)-\frac{X_{2}L_{1}a_{2}}{GI_{t1}}$$

The deformation at any position of S3 can be expressed as:

(64) $$d_{3}(a_{3})=\sum \int_{L_{i}}\frac{M_{i}(x_{i})\cdot {\bar{M}}_{Fi}(x_{i})dx_{i}}{EI_{i}}+\sum \int_{L_{i}}\frac{T_{i}(x_{i})\cdot {\bar{T}}_{Fi}(x_{i})dx_{i}}{GI_{ti}}$$

where,

(65) $${\bar{M}}_{F1}\left(x_{1}\right)=a_{3}-L_{1}+x_{1}$$

(66) $${\bar{T}}_{F1}\left(x_{1}\right)=-L_{2}$$

(67) $${\bar{M}}_{F2}\left(x_{2}\right)=-\left(L_{2}-x_{2}\right)$$

(68) $${\bar{T}}_{F2}\left(x_{2}\right)=-a_{3}$$

(69) $${\bar{M}}_{F3}\left(x_{3}\right)=-\left(a_{3}-x_{3}\right)$$

(70) $${\bar{T}}_{F3}\left(x_{3}\right)=0$$

where $a_{3}$ is a position indicator variable, $0\leq a_{3}\leq L_{3}$.

Assuming that:

(71) $$A_{1}(a_{3})=\int_{L_{1}}\frac{M_{1}(x_{1})\cdot {\bar{M}}_{F1}(x_{1})dx_{1}}{EI_{1}}=\frac{1}{EI_{1}}\left(-\frac{q_{1}L_{1}{}^{3}a_{3}}{6}+\frac{q_{1}L_{1}{}^{4}}{24}-\frac{X_{1}L_{1}{}^{2}a_{3}}{2}+\frac{X_{1}L_{1}{}^{3}}{6}+X_{3}L_{1}a_{3}-\frac{X_{3}L_{1}{}^{2}}{2}\right)$$

(72) $$A_{2}(a_{3})=\int_{L_{2}}\frac{M_{2}(x_{2})\cdot {\bar{M}}_{F2}(x_{2})dx_{2}}{EI_{2}}=\frac{1}{EI_{2}}\left(\frac{q_{2}L_{2}{}^{4}}{24}+\frac{q_{1}L_{1}L_{2}{}^{3}}{6}+\frac{X_{1}L_{2}{}^{3}}{6}-\frac{X_{2}L_{2}{}^{2}}{2}\right)$$

(73) $$\begin{array}{ll} A_{3}(a_{3}) & =\int_{a_{3}}\frac{M_{3}(x_{3})\cdot {\bar{M}}_{F3}(x_{3})dx_{3}}{EI_{3}}\\ & =\frac{1}{EI_{3}}\left(\frac{q_{3}a_{3}{}^{4}}{24}+\frac{q_{2}L_{2}a_{3}{}^{3}}{6}-\frac{q_{1}L_{1}{}^{2}a_{3}{}^{2}}{4}+\frac{q_{1}L_{1}a_{3}{}^{3}}{6}-\frac{X_{1}L_{3}a_{3}{}^{2}}{2}+\frac{X_{1}a_{3}{}^{3}}{6}+\frac{X_{3}a_{3}{}^{2}}{2}\right) \end{array}$$

(74) $$B_{1}(a_{3})=\int_{L_{1}}\frac{T_{1}(x_{1})\cdot {\bar{T}}_{F1}(x_{1})dx_{1}}{GI_{t1}}=-\frac{X_{2}L_{2}L_{1}}{GI_{t1}}$$

(75) $$B_{2}(a_{3})=\int_{L_{2}}\frac{T_{2}(x_{2})\cdot {\bar{T}}_{F2}(x_{2})dx_{2}}{GI_{t2}}=\frac{1}{GI_{t2}}\left(-\frac{q_{1}L_{1}{}^{2}L_{2}a_{3}}{2}-X_{1}L_{1}L_{2}a_{3}+X_{3}L_{2}a_{3}\right)$$

(76) $$B_{3}(a_{3})=\int_{a_{3}}\frac{T_{3}(x_{3})\cdot {\bar{T}}_{F3}(x_{3})dx_{3}}{GI_{t3}}=0$$

then,

(77) $$d_{3}\left(a_{3}\right)=A_{1}\left(a_{3}\right)+A_{2}\left(a_{3}\right)+A_{3}\left(a_{3}\right)+B_{1}\left(a_{3}\right)+B_{2}\left(a_{3}\right)+B_{3}\left(a_{3}\right)$$

The suspended inductor with auxiliary pillar is divided into two parts by the pillar, and each part contains three wire segments. The deformation and Von Mises stress of both parts can be calculated using the method described in this section.

## 3. Results and Discussion

This section describes the use of the ANSYS finite-element (FE) software to verify the calculated deformation results and the Von Mises stress of the suspended inductor with auxiliary pillar. The mechanical parameters of copper we used in FE analysis are: density $\rho =8900\;\mathrm{kg}/m^{3}$, Young’s modulus *E* = 128 GPa, Poisson’s ratio 0.34, and yield strength 100 MPa [20]. The inductor was meshed with tetrahedral mesh. The element size was 5 μm and the inductor model was divided into 4326 elements. To obtain the natural frequency of each part of the inductor coil, modal analyses of both parts were carried out. Table 1 lists the first three modal frequencies.

The frequency of the shock pulse in the actual environment was only on the order of 10^2^–10^4^ Hz, which is much smaller than the modal frequencies of the structures [13]. Hence, only the first modal frequency was taken into consideration.

**Figure 8:**
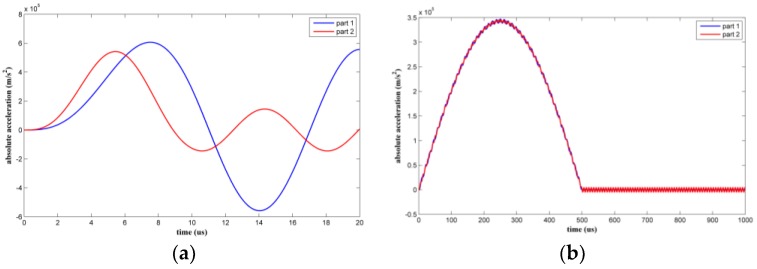
Absolute acceleration response of each part of the suspended inductor with auxiliary pillar: (**a**) τ = 10 μs, (**b**) τ = 500 μs.

Two types of shock pulse with an amplitude of 35,000 g were applied to the suspended inductor with auxiliary pillar. The duration of the first type was 10 μs, which is comparable to the vibration time period of the structure. The duration of the second type was 500 μs, which significantly exceeded the vibration time period of the structure. Figure 8 shows the absolute acceleration responses of the two coil parts.

When the shock load duration was 10 μs, the structure experienced the shock pulse as a dynamic load. The maximum absolute accelerations were amplified to various degrees. When the shock load duration was 500 μs, the structure experienced the shock pulse as a quasi-static load.

**Figure 9:**
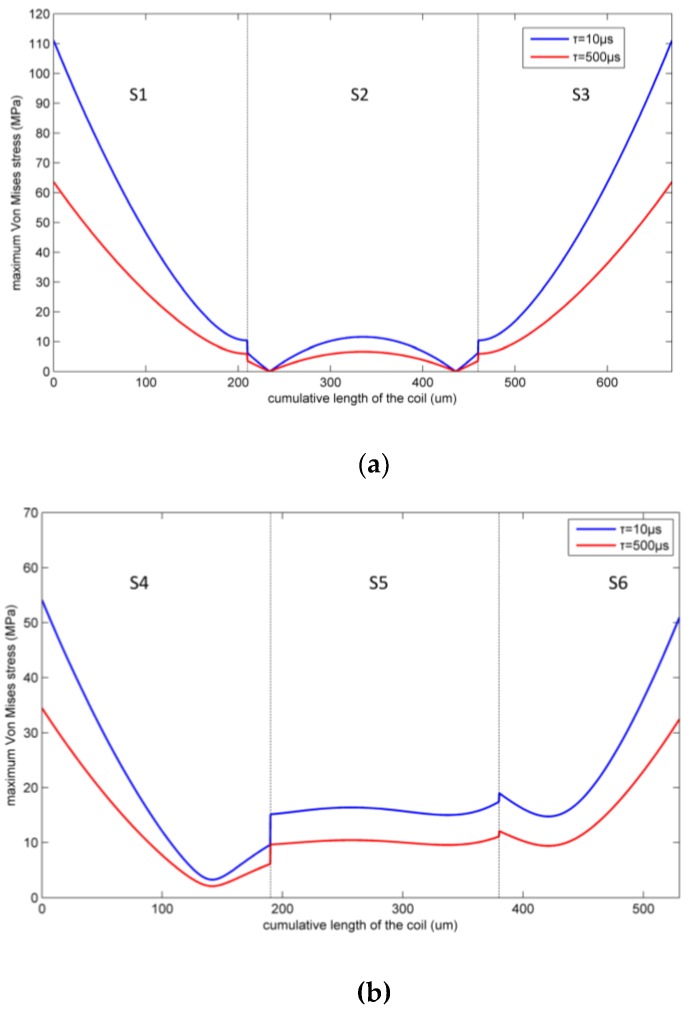
Maximum Von Mises equivalent stress of the inductor under two kinds of shock pulse. (**a**) Part 1; (**b**) Part 2.

As the height of the pillar is 20 μm and the maximum deformation of the pillar is only in 10^−3^ micron dimension according to the simulation results, we assume that the pillar does not bend under shock. We analyze the two coil parts separately. Figure 9 shows the maximum Von Mises equivalent stresses of the two inductor coil parts under the two kinds of shock.

The maximum Von Mises equivalent stress at any position on the inductor coil under a 10 μs duration shock was greater than under a 500 μs duration shock because the absolute acceleration of the coil parts under the 10 μs duration shock was higher. The maximum coil stress appeared at the two ends of Part 1 of the coil. As the shock load amplitude increases, plastic deformation of the inductor coil will occur first at these two positions.

**Figure 10:**
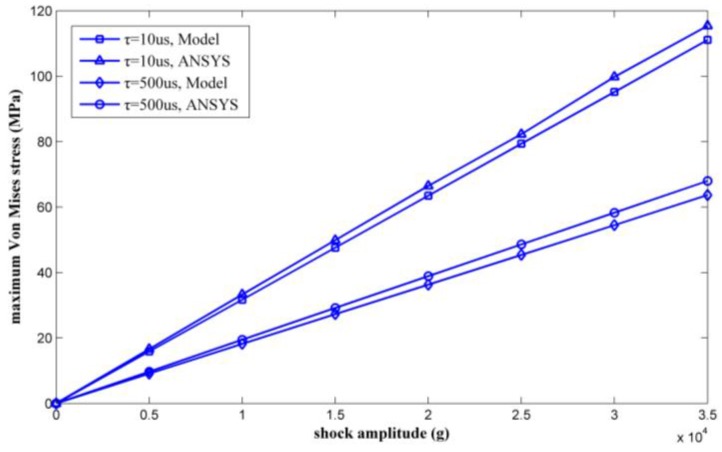
Maximum Von Mises equivalent stress by ANSYS and the model versus shock load amplitude.

By changing the shock load amplitude from 0 g to 35,000 g, a series of maximum Von Mises equivalent stresses of the inductor coil can be calculated. Figure 10 shows the calculated results as verified by ANSYS.

The maximum Von Mises stress is proportional to the amplitude of the shock pulse. The results calculated by the proposed model agree with the ANSYS simulation results. Plastic deformation will occur when the inductor undergoes a 30,000 g shock load with a duration of 10 μs, which occurred because the maximum Von Mises equivalent stress exceeded the yield strength of the copper (100 MPa in [20]). The maximum stress of the inductor coil under 35,000 g shock was solved for by the model proposed in this paper and was also simulated by ANSYS. Table 2 lists the results. The results achieved by ANSYS are considered as true values. The relative deviations of the maximum stress were 3.73% and 6.32% with shock durations of 10 μs and 500 μs, respectively.

**Figure 11:**
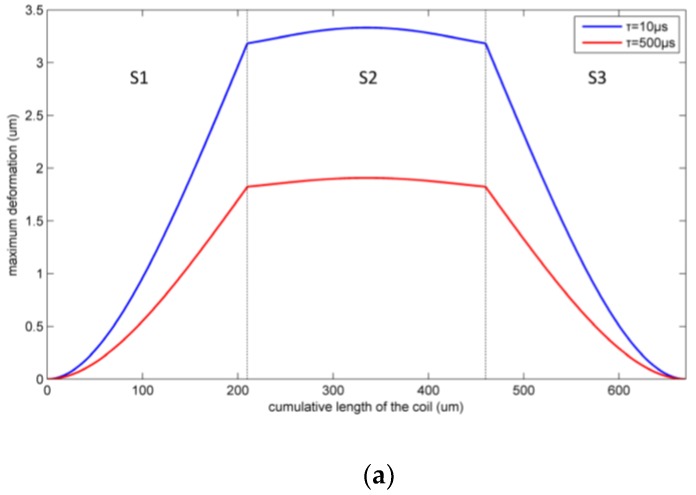

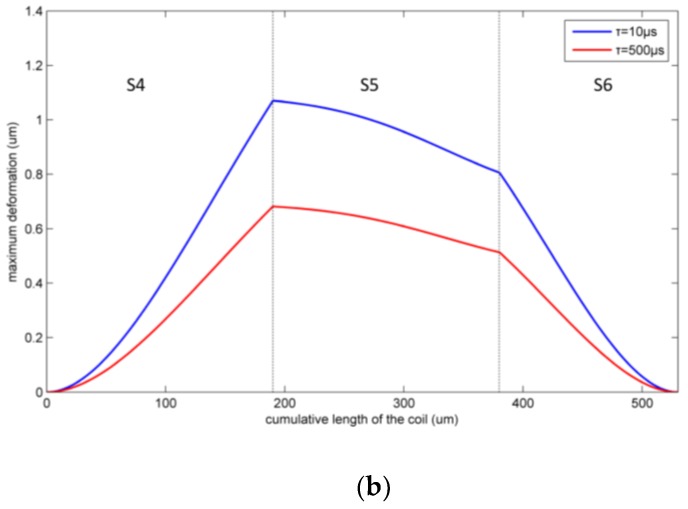
Maximum inductor deformation under two kinds of shock pulses. (**a**) Part 1; (**b**) Part 2.

Figure 11 shows the maximum deformation of the two inductor coil parts under the two kinds of shock pulses. The maximum inductor deformation appears at the midpoint of the S2 segment. As the shock load amplitude increases, the coil will first contact the substrate at this position due to the large deflection.

However, when a 35,000 g amplitude shock load with a duration of 10 μs is applied, the maximum deformation is only 3.33 μm, which is less than the distance between the coil and the lead. The coil will not strike to the lead or substrate when plastic deformation occurs. So the critical stress is the criterion for the failure of the suspended inductor with auxiliary pillar under shock.

**Figure 12:**
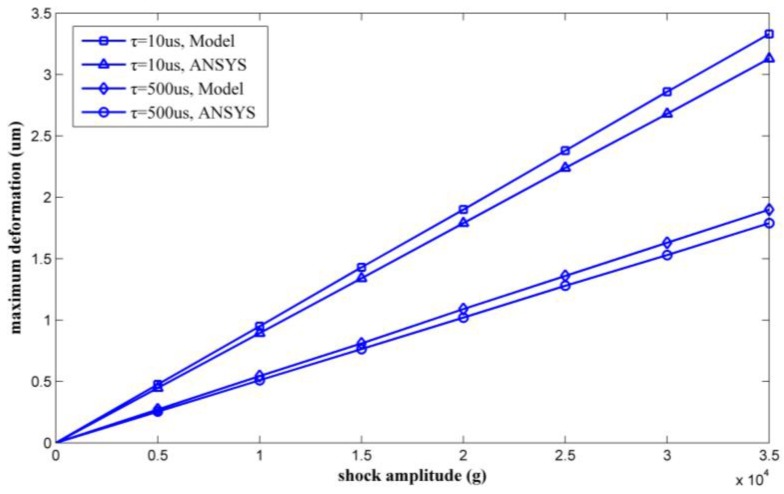
Maximum deformations as calculated by ANSYS and the proposed model versus shock load amplitude.

By varying the shock amplitude from 0 g to 35,000 g, a series of maximum deformations of the inductor coil can be calculated. Figure 12 shows the calculated results as verified by ANSYS.

The maximum deformation was also proportional to the amplitude of the shock pulse. The results calculated by the model agree with the ANSYS simulation results. The maximum coil deformations under 35,000 g shock were solved for by the model proposed in this paper and were also simulated by ANSYS. Table 3 lists the results. The relative deviations of the maximum deformation were 6.38% and 6.14% with shock durations of 10 μs and 500 μs, respectively.

Comparing the results obtained by theoretical calculation and ANSYS simulation, it was found that the results for maximum Von Mises equivalent stress and deformation calculated by the method proposed in this paper agreed with the results obtained by simulation, with relative deviations of less than 6.5%.

## 4. Extension of the Theory to the Suspended Inductor with Double Auxiliary Pillars

### 4.1. Mechanical Response of the Suspended Inductor with Double Auxiliary Pillars

**Figure 13:**
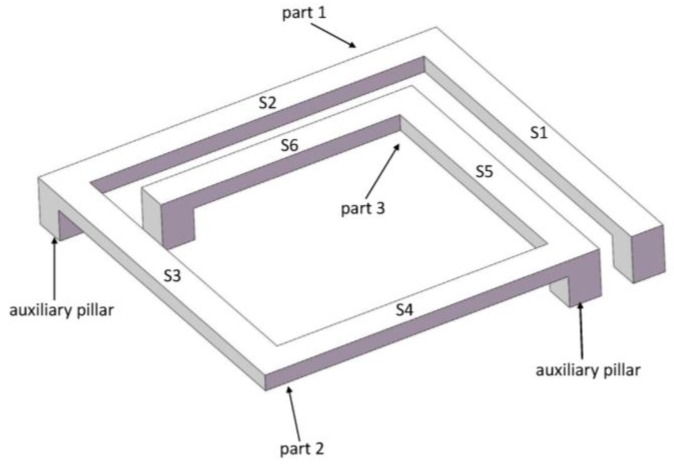
Schematic of MEMS suspended inductor with double auxiliary pillars.

In the work described in this section, the method proposed in this paper was extended to the suspended inductor with double auxiliary pillars, as shown in Figure 13. The suspended inductor with double auxiliary pillars has better mechanical performance than the inductor with only one pillar.

**Figure 14:**
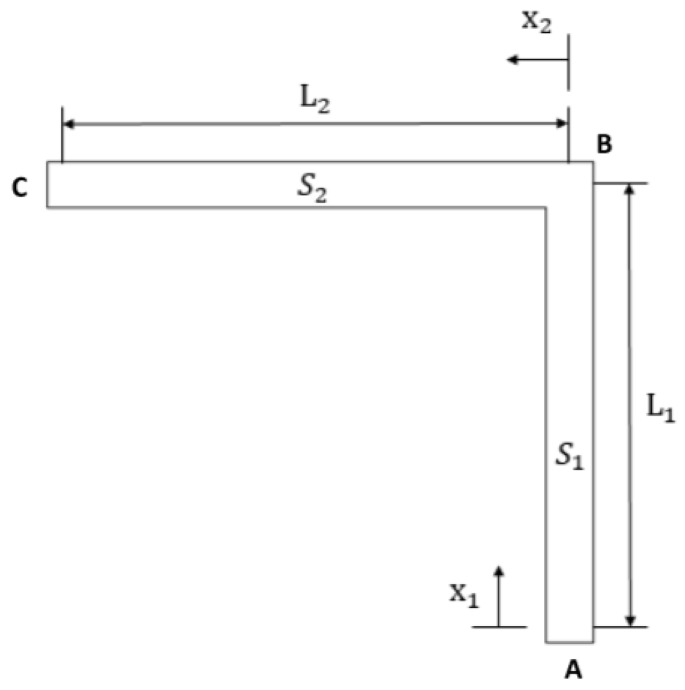
Top view of Part 1 of the coil.

The inductor coil is divided into three L-shaped bending beams by the pillars. Figure 14 shows a top view of Part 1.

The Von Mises equivalent stress and the deformation of the suspended inductor with double auxiliary pillars can be calculated by the following steps. First, the absolute acceleration response and the inertial distribution load of the L-shaped beam are calculated using Equations (5) and (6), respectively. $M_{qi}\left(x_{i}\right)$, $T_{qi}\left(x_{i}\right)$, ${\bar{M}}_{Fi}\left(x_{i}\right)$, ${\bar{T}}_{Fi}\left(x_{i}\right)$, ${\bar{M}}_{Ti}\left(x_{i}\right)$, ${\bar{T}}_{Ti}\left(x_{i}\right)$, ${\bar{M}}_{Mi}\left(x_{i}\right)$, and ${\bar{T}}_{Mi}\left(x_{i}\right)$ can be calculated using Equations (10)–(17), (24)–(27), and (32)–(35). Then $\delta_{ij}$ and $\Delta_{iF}$ can be obtained by Mohr’s integration. By substituting $\delta_{ij}$ and $\Delta_{iF}$ into the canonical Equation (7), the generalized forces $X_{1}$, $X_{2}$, and $X_{3}$ can be obtained. The bending moment and the torque of the two wire segments can be calculated using Equations (45)–(48).

The Von Mises equivalent stress of the *i*-th wire segment can be expressed as:

(78) $$\sigma_{i-VonMises}\left(x_{i}\right)=\sqrt{{\left(\frac{M_{i}\left(x_{i}\right)}{I_{i}}\cdot \frac{t_{i}}{2}\right)}^{2}+3{\left(\frac{T_{i}\left(x_{i}\right)}{\alpha_{i}w_{i}t_{i}{}^{2}}\right)}^{2}}$$

The deformation along the normal direction of the coil plane at any position along the wire segment S1 can be expressed by:

(79) $$d_{1}\left(a_{1}\right)=\frac{1}{EI_{1}}\left(\frac{q_{1}a_{1}{}^{4}}{24}+\frac{X_{1}a_{1}{}^{3}}{6}-\frac{X_{3}a_{1}{}^{2}}{2}\right)$$

The deformation along the normal direction of the coil plane at any position along the wire segment S2 can be expressed by:

(80) $$d_{2}\left(a_{2}\right)=\frac{1}{EI_{1}}\left(\frac{q_{1}L_{1}{}^{4}}{24}+\frac{X_{1}L_{1}{}^{3}}{6}-\frac{X_{3}L_{1}{}^{2}}{2}\right)+\frac{1}{EI_{2}}\left(\frac{q_{2}a_{2}{}^{4}}{24}+\frac{q_{1}L_{1}a_{2}{}^{3}}{6}+\frac{X_{1}a_{2}{}^{3}}{6}-\frac{X_{2}a_{2}{}^{2}}{2}\right)-\frac{X_{2}L_{1}a_{2}}{GI_{t1}}$$

where $a_{1}$ and $a_{2}$ are position indicator variables, $0\leq a_{1}\leq L_{1}$, $0\leq a_{2}\leq L_{2}$.

For other kinds of inductors with auxiliary pillars, we assume that the inductor coil is divided into *n* + 1 parts by *n* pillars and each part contains *m* wire segments. We can calculate the Von Mises equivalent stress and deformation of each coil part by the following steps:

1. Obtain the natural frequency of the coil part and calculate the acceleration response of the coil part by using a SDOF model.
2. Each coil part is a statically indeterminate structure, so its equivalent system can be determined by releasing the constraint of one of its clamped end. Then the canonical equation of the coil part can be established as:
  (81) $$\left[\begin{array}{lll} \delta_{11} & \delta_{12} & \delta_{13}\\ \delta_{21} & \delta_{22} & \delta_{23}\\ \delta_{31} & \delta_{32} & \delta_{33} \end{array}\right]\cdot \left[\begin{array}{l} X_{1}\\ X_{2}\\ X_{3} \end{array}\right]+\left[\begin{array}{l} \Delta_{1F}\\ \Delta_{2F}\\ \Delta_{3F} \end{array}\right]=\left[\begin{array}{l} \Delta_{1}\\ \Delta_{2}\\ \Delta_{3} \end{array}\right]$$
  where $\delta_{ij}$ and $\Delta_{iF}$ can be expressed as Equations (82) and (83), respectively, according to Mohr’s integration:
  (82) $$\delta_{ij}=\overset{m}{\underset{k=1}{\sum }}\int \frac{{\bar{M}}_{jk}{\bar{M}}_{ik}}{EI_{k}}dx_{k}+\overset{m}{\underset{k=1}{\sum }}\int \frac{{\bar{T}}_{jk}{\bar{T}}_{ik}}{GI_{tk}}dx_{k}$$
  (83) $$\Delta_{iF}=\overset{m}{\underset{k=1}{\sum }}\int \frac{M_{qk}{\bar{M}}_{ik}}{EI_{k}}dx_{k}+\overset{m}{\underset{k=1}{\sum }}\int \frac{T_{qk}{\bar{T}}_{ik}}{GI_{tk}}dx_{k}$$
  where $M_{qk}$ and $T_{qk}$ are the bending moment and torque of the *k*-th wire segment caused by the impact load, respectively. ${\bar{M}}_{ik}$ and ${\bar{T}}_{ik}$ are the bending moment and torque of the *k*-th wire segment caused by the unit load along the $X_{i}$ direction at the released end, respectively. ${\bar{M}}_{jk}$ and ${\bar{T}}_{jk}$ are the bending moment and torque of the *k*-th wire segment caused by the unit load along the $X_{j}$ direction at the released end, respectively.
3. The generalized forces $X_{1}$, $X_{2}$ and $X_{3}$ can be obtained by solving the canonical equation. Then the bending moment $M_{k}$ and the torque $T_{k}$ of the *k*-th wire segment can be calculated.
4. Calculate the critical normal stress $\sigma_{k-max}$ and the critical shear stress $\tau_{k-max}$ of the *k*-th wire segment. Then the Von Mises equivalent stress of the *k*-th wire segment can be expressed as:
  (84) $$\sigma_{k-VonMises}=\sqrt{\sigma_{k-max}{}^{2}+3\tau_{k-max}{}^{2}}$$
5. Apply a unit force to the position wherever the deformation needs to be solved and adopt the Mohr’s integration for calculating the deformation of the coil part. The deformation at any position of the *k*-th wire segment can be expressed as:
  (85) $$d\left(a_{k}\right)=\overset{k-1}{\underset{i=1}{\sum }}\int_{0}^{L_{i}}\frac{M_{i}{\bar{M}}_{Fi}}{EI_{i}}dx_{i}+\overset{k-1}{\underset{i=1}{\sum }}\int_{0}^{L_{i}}\frac{T_{i}{\bar{T}}_{Fi}}{GI_{ti}}dx_{i}+\int_{0}^{a_{k}}\frac{M_{k}{\bar{M}}_{Fk}}{EI_{k}}dx_{k}+\int_{0}^{a_{k}}\frac{T_{k}{\bar{T}}_{Fk}}{GI_{tk}}dx_{k}$$
  where $M_{i}$ and $M_{k}$ are the bending moments of the *i*-th and *k*-th wire segment, respectively. $T_{i}$ and $T_{k}$ are the torques of the *i*-th and *k*-th wire segment, respectively. ${\bar{M}}_{Fi}$ and ${\bar{M}}_{Fk}$ are the bending moments of the *i*-th and *k*-th wire segment caused by the unit force, respectively. ${\bar{T}}_{Fi}$ and ${\bar{T}}_{Fk}$ are the torques of the *i*-th and *k*-th wire segment caused by the unit force, respectively. $a_{k}$ is a position indicator variable, $0\leq a_{k}\leq L_{k}$.

### 4.2. Results and Discussion

Modal analyses of the three coil parts were carried out. The inductor with double auxiliary pillars was also meshed with tetrahedral mesh. The element size was 5 μm and the inductor model was divided into 4739 elements. The natural frequencies of the three parts were found to be 0.17 MHz, 0.21 MHz, and 0.33 MHz, respectively.

**Figure 15:**
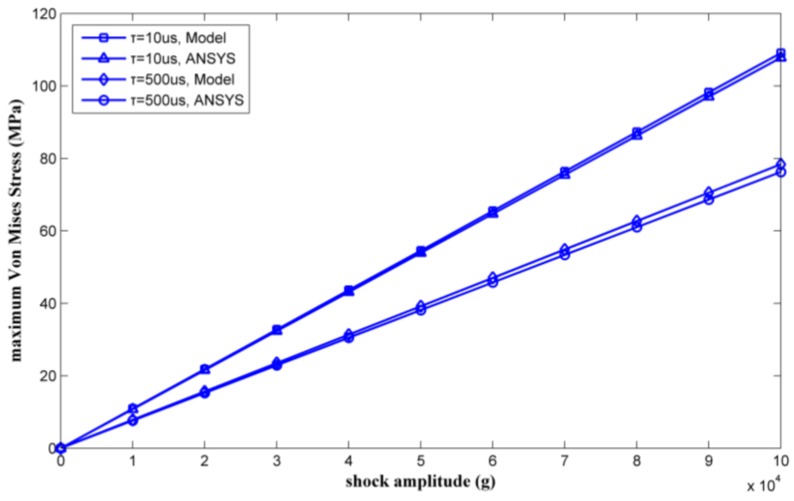
Maximum Von Mises equivalent stress as calculated by ANSYS and the proposed model versus shock load amplitude.

**Figure 16:**
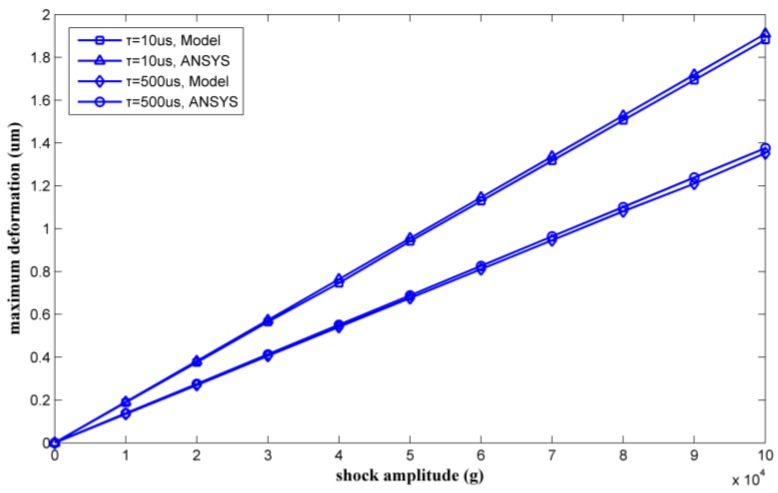
Maximum deformation as calculated by ANSYS and the proposed model versus shock load amplitude.

Two types of shock pulse were applied to the inductor. The durations of the shock pulses were 10 μs and 500 μs. By varying the shock load amplitude from 0 g to 100,000 g, a series of results for the maximum Von Mises equivalent stress and the maximum deformation of the inductor coil could be calculated. The results obtained by the method proposed in this paper were verified by ANSYS, as shown in Figure 15 and Figure 16.

Figure 15 and Figure 16 show that the results obtained by the method proposed in this paper agreed well with the results obtained by ANSYS simulation. The mechanical performance of the suspended inductor with double auxiliary pillars has been significantly improved.

The maximum stresses and the maximum deformations of the inductor under 100,000 g shock load obtained by the method proposed in this paper and by ANSYS are listed in Table 4 and Table 5, respectively.

Comparing the results obtained by theoretical calculation and ANSYS simulation, it is apparent that the calculated results agree well with the simulated results, with relative deviations of less than 3.1%.

**Figure 17:**
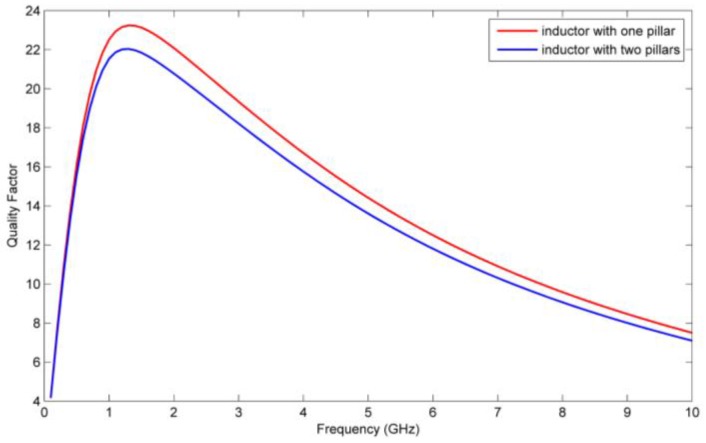
Quality factors of the inductors with single and double auxiliary pillar(s).

**Figure 18:**
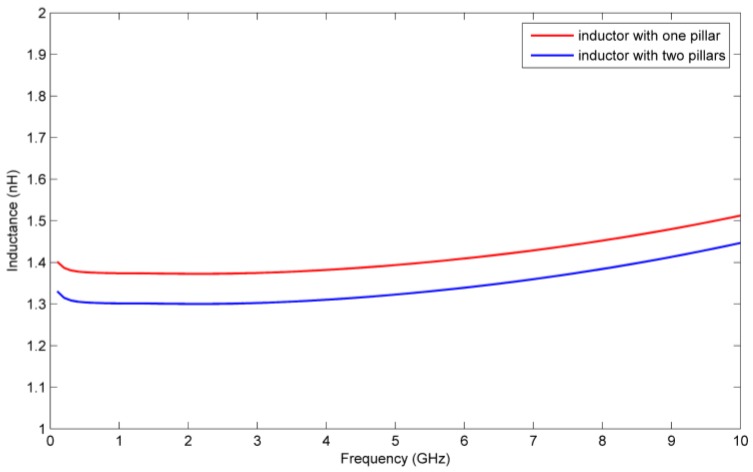
Inductances of the inductors with single and double auxiliary pillar(s).

Besides the mechanical performance, the radio-frequency performance of the inductors with one and double pillar(s) are also considered. The RF performance of the inductor with single and double pillar(s) are simulated by using the electromagnetic finite element analysis software HFSS. The quality factors and inductances of the two kinds of inductors are shown in Figure 17 and Figure 18, respectively.

From Figure 17 and Figure 18 we see that the quality factor and the inductance of the inductor with single pillar are only 1.2 and 0.07 nH higher, respectively, than the inductor with double pillars. Although the inductor with double pillars has a worse RF performance because one more copper pillar leads to an increase in the substrate loss, its mechanical performance is much better than the inductor with a single pillar.

## 5. Conclusions

In this paper, a method has been proposed to describe the response of MEMS suspended inductor with auxiliary pillar under high mechanical shock by combining an SDOF model and a method of solving statically indeterminate structures. This method was used to calculate the equivalent stress and the deformation of the suspended inductor with auxiliary pillars under high mechanical shock. The calculated results were found to agree well with the simulation results. The stress and deformation of other types of inductors with auxiliary pillar can also be calculated using this method. The method proposed in this paper provides a theoretical reference for mechanical performance evaluation and reliability optimization design of MEMS suspended inductors with auxiliary pillars.

## Figures and Tables

**Table 1:** First three modal frequencies of each part of the inductor with auxiliary pillar.

Part Number	Modal 1 Frequency/MHz	Modal 2 Frequency/MHz	Modal 3 Frequency/MHz
1	0.083042	0.18326	0.26661
2	0.13383	0.31126	0.44392

**Table 2:** Maximum Von Mises equivalent stress of the inductor with auxiliary pillar as calculated by ANSYS and the proposed model.

Amplitude of Shock/g	Duration of Shock/μs	Max Von Mises Stress by Model/MPa	Max Von Mises Stress by ANSYS/MPa	Relative Deviation
35,000	10	111.1	115.4	3.73%
	500	63.7	68	6.32%

**Table 3:** Maximum deformation of the inductor with auxiliary pillar as calculated by ANSYS and the proposed model.

Amplitude of Shock/g	Duration of Shock/μs	Max Deformation by Model/μm	Max Deformation by ANSYS/μm	Relative Deviation
35,000	10	3.33	3.13	6.38%
	500	1.90	1.79	6.14%

**Table 4:** Maximum Von Mises equivalent stresses of the inductor with double auxiliary pillars as calculated by ANSYS and the proposed model.

Amplitude of Shock/g	Duration of Shock/μs	Max Von Mises Stress by Model/MPa	Max Von Mises Stress by ANSYS/MPa	Relative Deviation
100,000	10	109.1	108.6	0.46%
	500	78.37	77.21	1.50%

**Table 5:** Maximum deformations of the inductor with double auxiliary pillars, as calculated by ANSYS and the proposed model.

Amplitude of Shock/g	Duration of Shock/μs	Max Deformation by Model/μm	Max Deformation by ANSYS/μm	Relative Deviation
100,000	10	1.88	1.94	3.09%
	500	1.35	1.38	2.17%
